# Relationship between Biodegradation Rate and Grain Size Itself Excluding Other Structural Factors Caused by Alloying Additions and Deformation Processing for Pure Mg

**DOI:** 10.3390/ma15155295

**Published:** 2022-08-01

**Authors:** Zhan Qu, Lulin Liu, Youming Deng, Ran Tao, Weidong Liu, Zhongren Zheng, Ming-Chun Zhao

**Affiliations:** 1 Xiangya Hospital, Central South University, Changsha 410008, China; quzhan@csu.edu.cn (Z.Q.); dymkingdom@csu.edu.cn (Y.D.); trxyyy@csu.edu.cn (R.T.); 2 International Joint Research Center of Minimally Invasive Endoscopic Technology Equipment & Standards, Changsha 410008, China; 3 Shanghai East Hospital, Dalian Medical University, Shanghai 201200, China; csullliu@csu.edu.cn; 4 School of Materials Science and Engineering, Central South University, Changsha 410083, China; csuzrzheng@csu.edu.cn (Z.Z.); mczhao@csu.edu.cn (M.-C.Z.)

**Keywords:** pure Mg, implant metals, biodegradation, grain size, Hall–Petch relation

## Abstract

This work studied the relationship between biodegradation rate and grain size itself, excluding other structural factors such as segregations, impure inclusions, second phase particles, sub-structures, internal stresses and textures caused by alloying additions and deformation processing for pure Mg. A spectrum of grain size was obtained by annealing through changing the annealing temperature. Grain boundary influenced the hardness and the biodegradation behavior. The hardness was grain size-dependent, following a typical Hall–Petch relation: $\mathrm{HV}=18.45+92.31d^{-}{}^{\frac{1}{2}}$. The biodegradation rate decreased with decreasing grain size, following a similar Hall–Petch relation: $P_{i}=0.17-0.68d^{-}{}^{\frac{1}{2}}$ or $P_{w}=1.34-6.17d^{-}{}^{\frac{1}{2}}$. This work should be helpful for better controlling biodegradation performance of biodegradable Mg alloys through varying their grain size.

## 1. Introduction

In recent years, Mg and its alloys have attracted much attention as biomedical implant materials due to their mechanical compatibility, biocompatibility and biodegradability [1,2,3,4]. Grain size is an important trait influencing the biodegradation rate in physiological environments for biodegradable biomedical metals [5,6,7]. The finer grain size has more grain boundary. Grain boundaries and interiors present different properties in terms of atomic reactivity, diffusion rate and coordination. The biodegradation of implant metals in body fluid environments first occurs on the surface. Therefore, the finer grained surface is thought to have different electrochemical behavior and accordingly has different biodegradation rate from coarser grained surface. Many studies indicated that for pure Mg and its alloys, a finer grain size led to a lower biodegradation rate [8,9,10]. 

However, (i) most work to date has only studied coarse- and fine-grained alloys (and not a spectrum of grain size); and (ii) grain refinement was usually achieved through alloying additions and/or deformation processing. Alloying additions may cause segregations, impure inclusions and second phase particles. Deformation processing may cause sub-structures, internal stresses and textures. Therefore, alloying additions and/or deformation processing can not exclude other structural factors such as segregations, impure inclusions, second phase particles, sub-structures, internal stresses and textures, in addition to the modification of the intentional grain size. Such secondary consequences may be involved in these other structure factors, each of which may have an impact on the degradation response. It makes the relationship between the grain size and the biodegradation rate difficult to extract. For example, according to classical electrochemical corrosion theory, second phase particles caused by the alloying additions might form a micro-galvanic corrosion with the matrix to accelerate the degradation [11]. Therefore, the relationship between grain size and biodegradation rate is inherently complex. It is very necessary to study the relationship between biodegradation rate and grain size, excluding these other structural factors. 

In this work, in order to exclude the effects of these other structural factors on biodegradation rate to the maximum extent, pure Mg was used, for which homogeneous microstructures with a spectrum of grain size were obtained by annealing through changing the annealing temperature. The role of grain size on biodegradation rate was investigated. The biodegradation rate was characterized by electrochemical polarization curve and impedance spectrum combined with immersion test. To our best knowledge, never before has the work to deliberately consider the effect of grain size itself to exclude the effects of other structural factors from the alloying additions and/or the deformation processing on biodegradation rate. From this aspect, the obtained relationship that exists between degradation rate and grain size is, to some degree, a novelty and is different from those published literatures. This work helps to clarify the relationship between biodegradation rate and grain size itself excluding other structural factors caused by alloying additions and deformation processing for pure Mg. It also provides a reference for grain size modification to decrease the biodegradation rate of pure Mg and facilitate its use in practical applications.

## 2. Experimental Procedures

Pure Mg was used in the present work, having chemical compositions (wt.%) of 0.002 Fe, 0.003 Si, 0.0004 Ni, 0.0005 Cu, 0.004 Al, 0.005 Mn and Mg balance, which is of high purity and is thus suitable for the study of the influences of grain size on biodegradation, other than the effect of the impurity elements. 

The specimens with a diameter of 10 mm and a length of 3 mm were cut from the rods that were extruded from the cast ingots of pure Mg produced as a laboratory-scale vacuum-melted ingot. Annealing was performed for the extruded specimens at 350 °C, 400 °C, 450 °C and 500 °C for 120 min followed by air cooling. Hereafter, these four annealed conditions were designed as Mg350, Mg400, Mg450, Mg500, respectively. The grain size was observed using optical microscopy by etching the polished surfaces of the specimens with a 2% nitric acid alcohol. The hardness was measured by MC010-HVS-1000 digital Vickers indenter.

Potentiodynamic polarization tests were performed in Hank’s solution with the following compositions: NaCl 8.0 g/L, D-Glucose 1.0 g/L, KCl 0.4 g/L, NaHCO_3_ 0.35 g/L, CaCl_2_ 0.14 g/L, MgSO_4_·7H_2_O 0.2 g/L, Na_2_HPO_4_·12H_2_O 0.12 g/L, KH_2_PO_4_ 0.06 g/L at 37 ± 0.5 °C. Electrochemical tests were carried out using an Autolab system by using a conventional three electrodes system with a saturated calomel electrode (SCE) as the reference, a platinum electrode as the counter and the sample as the working electrode. The electrochemical impedance spectrum was tested after the open circuit potential was stable for 1800 s. The amplitude was 10 mV, and the test frequency range was 10^5^–10^−2^ Hz. After the EIS test was completed, the potentiodynamic polarization experiment was started at a scan rate of 0.5 mV/s at −250 mV below the open circuit potential (OCP). From the potentiodynamic polarization curves, the biodegradation rate, i.e., an average penetration rate, *P*_i_ (mm y^−1^), was evaluated from the corrosion current density, *i*_corr_ (mA cm^−2^), using the following conversions Equation (1) [12]:

*P*_i_ = 22.85 *i*_corr_ (1)

Immersion tests were conducted in Hank’s solution at 37 ± 0.5 °C for 14 days. The initial weight of specimens was recorded before immersing, and the solution was refreshed every 24 h. After immersion tests, the specimens were cleaned with chromium trioxide solution to remove the surface corrosion products, and then were weighted. The weight loss rate, Δ*W* (mg cm^−2^ d^−1^), and the corresponding biodegradation rate, *P*_w_ (mm y^−1^), were calculated using the formulas Equations (2) and (3) [12], respectively:

Δ*W* = (*W*_b_ − *W*_a_)/*AT* (2)

*P*_w_ = 3.67 Δ*W/D* (3)

where *W*_b_ and *W*_a_ are the specimen mass before exposure and after exposure, respectively, *A* is the specimen area exposed to the solution (cm^2^), *T* is the time (d), and *D* is the density of the material in g/cm^3^ (1.73 g/cm^2^ for pure Mg in this work). 

Corrosion morphologies and topographic maps after immersion tests were characterized using digital camera, SEM and 3D measuring laser microscope, respectively. 

## 3. Results

### 3.1. Grain Size and Hardness

**Figure 1:**
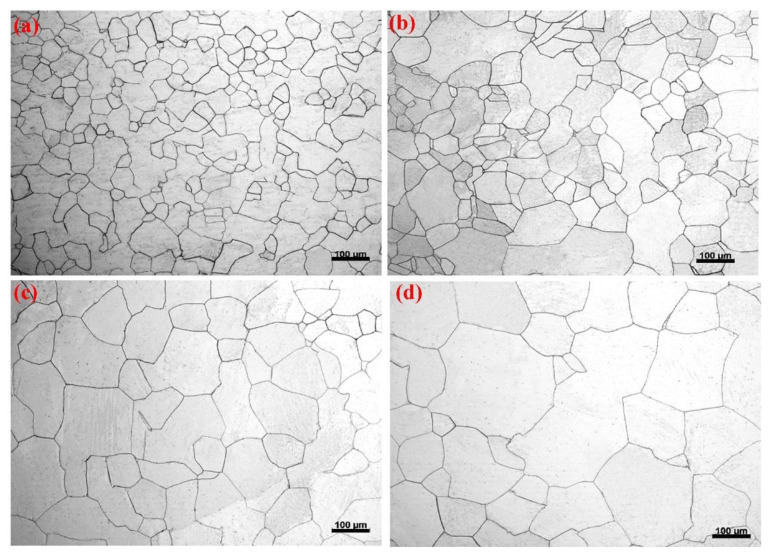
Optical micrographs showing the homogeneous microstructures with different grain size at different annealed conditions: (**a**) 350 °C, (**b**) 400 °C, (**c**) 450 °C, and (**d**) 500 °C.

Figure 1 shows the optical micrographs of the different annealed conditions, which presents homogeneous microstructures with different grain size. All the microstructures were composed of quai-polygonal or equiaxed grains having no apparent inclusion in the matrix. This indicated that the experimental pure Mg was clean. Therefore, the influence of the inclusions on the biodegradation behavior is expected to be minimal.

**Figure 2:**
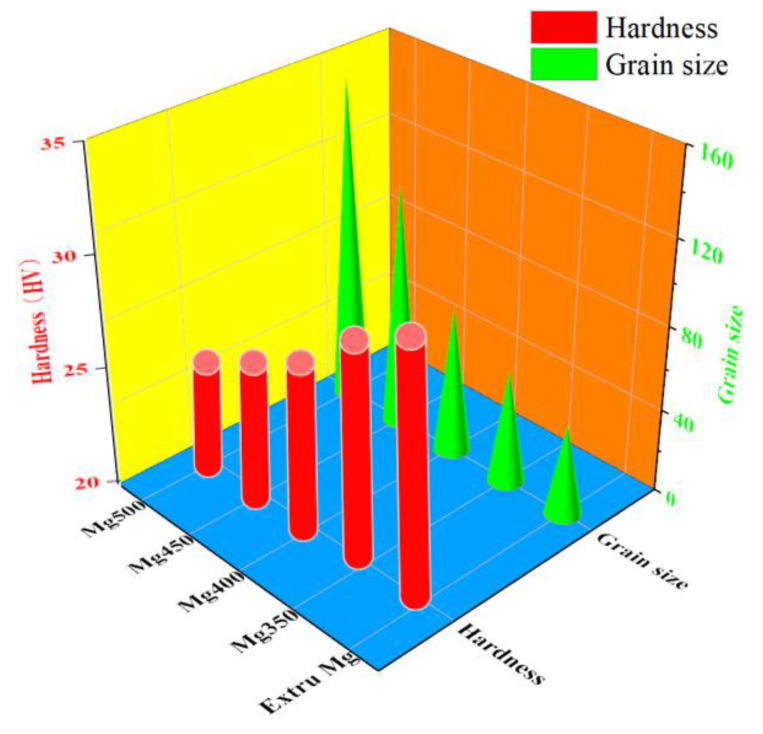
The average grain size and Vickers hardness at different annealed conditions (Mg350, Mg400, Mg450 and Mg500), with the extruded condition (Extru Mg) as a comparison.

Figure 2 shows the average grain size of the different annealed conditions. The average grain size was 44 μm for the initial extruded condition, which is also depicted in Figure 2. Extruded pure Mg essentially accumulated a lot of stored energy during deformation [13]. As annealed, nuclei were formed at the grain boundaries or subgrains of the initial deformed structure, and new undistorted grains that had little dislocation density and residual stress were regenerated, i.e., grain growth, as shown in Figure 2. There was a pronounced grain size evolution as the annealed schedules changed in temperature. When annealed at 350 °C, the average grain size was 55 μm. The increase of the annealed temperature resulted in a substantial coarsening of the grain size. When annealed at 400 °C, 450 °C and 500 °C, the average grain size was 71 μm, 117 μm and 155 μm, respectively. The driving force of recrystallization was larger at higher temperature, causing larger new undistorted grains. As mentioned above, the grain size grew to 155 μm when annealed at 500 °C, which was ~3 times larger than 44 μm of the extruded condition. The above results indicated that the driving force of recrystallization was larger at higher annealed temperature, causing the larger grain size. For example, the grain size grew to 155 μm when annealed at 500 °C, which was ~3.5 times larger than 44 μm of the initial extruded condition and ~2 times larger than 71 μm of the annealed condition at 400 °C. Figure 2 also shows the Vickers hardness of the above-mentioned different conditions. The hardness was 32.9 HV for the extruded condition, which was also depicted in Figure 2. There was a hardness change as the annealed schedules changed in temperature. When annealed at 350 °C, the hardness was 30.6 HV. With the increase of the annealing temperature, the hardness decreased. When annealed at 400 °C, 450 °C and 500 °C, the hardness was 28.7 HV, 27.4 HV and 25.9 HV, respectively.

**Figure 3:**
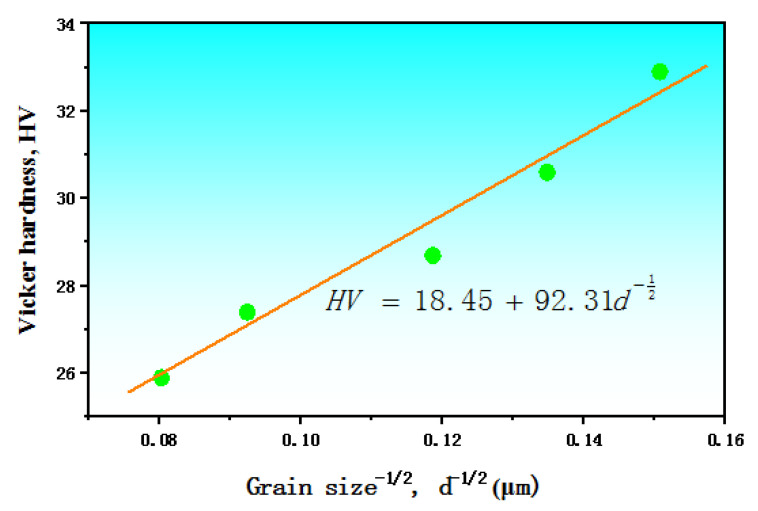
The relation between the Vickers hardness (HV) and the average ferrite size (*d*).

Figure 3 shows a plot between the Vickers hardness and the minus square root of the grain size, i.e., HV versus *d*^−1/2^, having a monotonic character. The grain size increased with the increase of the annealing temperature, and the hardness decreased with the increase of the annealing temperature; in other words, with the increase of the grain size, as shown in Figure 2. The grain boundaries hindered dislocation movement. The smaller grain size had the larger proportion of grain boundaries, resulting in a more obvious hindrance [14]. In addition, the undistorted grain obtained by recrystallization annealing could also reduce the hardness. The HV versus *d*^−1/2^ curve synchronized well. This indicated that the hardness of pure Mg was grain size-dependent, expressed by a single line that clearly followed a typical Hall–Petch relation [15]. A regression equation to describe the relationship between the hardness and the grain size was developed as Equation (4) with the correlation coefficient of 0.98, using all the experimental data.

(4) $$\mathrm{HV}=18.45+92.31d^{-}{}^{\frac{1}{2}}$$

From the originally expressed dependence of the yield stress on the grain size, the Hall–Petch relation was successfully applied to describe the dependence of the hardness on the grain size for pure Mg in this work, which implied a hardening potential by grain refinement for the pure Mg.

### 3.2. Biodegradation

**Figure 4:**
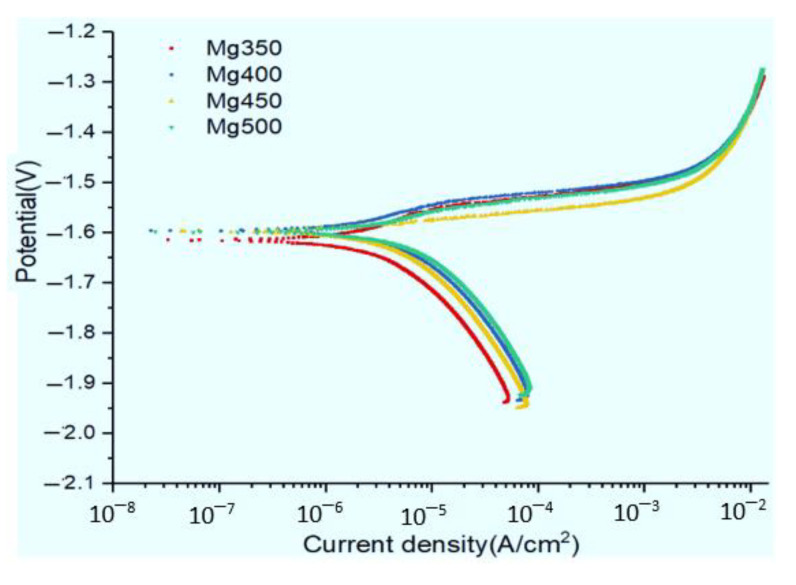
Polarisation curves of the different annealed conditions in Hank’s solution.

Figure 4 shows the potentiodynamic polarization curves of the different annealed conditions in the Hank’s solution. Different annealed conditions resulted in only a very lightly change in the corrosion potential (*E*_corr_) but a relatively obvious change in the *i*_corr_. The *i*_corr_ values evaluated from the polarization potential curves by Tafel extrapolation using the linear cathodic branch are listed in Table 1. The *i*_corr_ increased with increasing the annealed temperature; in other words, with increasing grain size. The biodegradation rate, *P*_i_, is also listed in Table 1, which was calculated from the *i*_corr_ using Equation (1). The biodegradation rate, *P*_i_, decreased with decreasing grain size.

**Figure 5:**
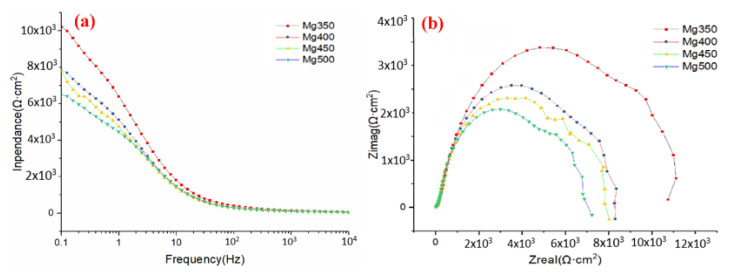
(**a**) Bode plots and (**b**) Nyquist plots of the different annealed conditions in Hank’s solution.

**Figure 6:**
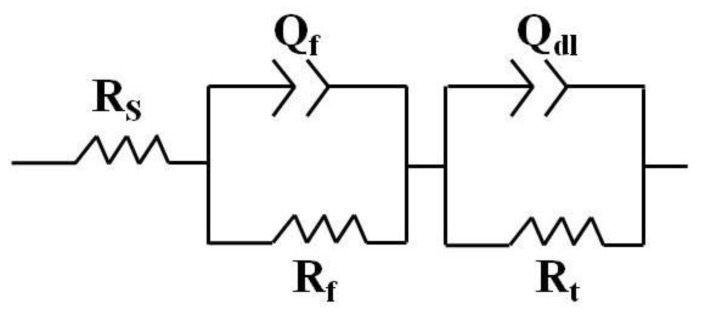
An equivalent circuit for impedance data fitting.

Figure 5 shows the electrochemical impedance spectra (EIS). The Nyquist plots had the same shape at different annealed temperatures, including one high frequency capacitance loop and one low frequency capacitance loop in the measured frequency range. Generally, a high frequency capacitance loop reflects the electrode dynamic process of the system, and low frequency capacitance loop reflects the mass transfer relaxation process of the biodegradation product layer on the surface. The diameter of the capacitive loop is associated with the polarization resistance. A larger diameter capacitive loop represents a lower biodegradation rate [16]. The size order of the diameter was decreased with increasing the annealed temperature, i.e., with increasing the grain size, as shown in Figure 5. In order to get more information from the EIS spectra, the equivalent circuit illustrated in Figure 6 was used to analyze the Nyquist spectra. The corresponding data are listed in Table 2, where R_s_ is solution resistance, R_t_ is charge transfer resistance, and CPE_dl_ is double-layer capacitor. R_f_ and CPE_f_ are film resistances and capacitance corresponding to the surface film effect [17,18], respectively. The order of the R_t_ values was Mg350 > Mg400 > Mg450 > Mg500, corresponding to the order of the EIS spectra diameter. 

**Figure 7:**
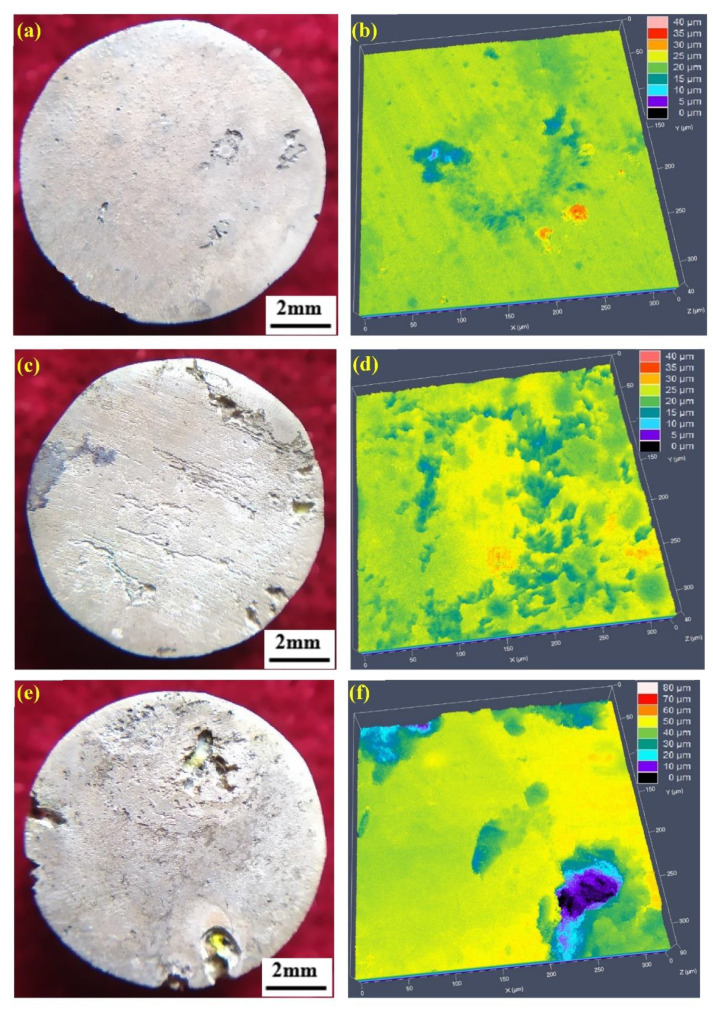
Surface morphologies (**a**,**c**,**e**) and topographic maps (**b**,**d**,**f**) of the representative annealed conditions: (**a**,**b**) for annealed at 350 °C, (**c**,**d**) for annealed at 450 °C, (**e**,**f**) for annealed at 500 °C.

Figure 7 shows the surface morphologies and the topographic maps of the representative annealed conditions after removing the biodegradation products, which have been immersed for 14 days in Hank’s solution. The surface morphologies and topographic maps were various for the different annealed conditions. When annealed at 350 °C, biodegradation was relatively more uniform on a macro-scale; there was little preferential sites for the biodegradation (Figure 7a). The corresponding topographic map was shown in Figure 7b, in which different biodegradation depths corresponded to different colors. The pits were small and shallow with diameters up to ~10 μm and depths up to ~15 μm. For other annealed temperatures of 450 °C and 500 °C, the surfaces had suffered relatively more severe biodegradation attack, which presented localized deep biodegradation areas, as shown Figure 7c,e. Their corresponding topographic maps are shown in Figure 7d,f, in which the pits are larger and deeper. The topographic maps were in agreement with the results of the corresponding surface morphologies. The biodegradation became more serious with increasing the annealed temperature, i.e., with increasing grain size. The biodegradation rate, *P*_w_, is also listed in Table 1, which is calculated from the weight loss using Equations (2) and (3). The biodegradation rate, *P*_w_, decreased with decreasing grain size. The biodegradation degree from the immersion tests including the observation of surface morphologies and topographic maps and the measurement of the biodegradation rate, *P*_w_, was in agreement with that from the above potentiodynamic polarization tests.

## 4. Discussion

The difference in biodegradation for different annealed conditions was mainly attributed to the change of grain size caused by annealing, because there was no influence of the second phase in pure Mg, and the designed annealing above recrystallization temperature was also enough to eliminate most of the residual stress. The change of grain size resulted in the change of grain boundary area. The grain boundary segregation was demonstrated to occur in pure Mg (99.9%) [19], pure iron (99.9%) [20], pure Mo (99.9%) [21], pure Cu (99.99%) [22] and pure Au (99.99%) [23]. Therefore, grain boundary influences the biodegradation behavior of pure metals. For pure Mg, the matrix acts as anode and grain boundary acts as cathode, because the standard potential of Mg matrix is very negative in metals, only −2.37 V, lower than almost any other metal, while the standard potential of the impurity segregations at grain boundary is much higher than that of Mg matrix. The smaller grain size presents the greater number of grain boundaries. In this case, a large number of potential corrected grain boundaries covered the pure Mg grains and encapsulated the pure Mg grains to form a biodegradation barrier. Therefore, the biodegradation resistance of pure Mg increased with the decrease of grain size. As shown in Table 1, the corrosion potential of pure Mg at different annealed temperatures hardly changed. Corrosion potential reflects the thermodynamic information of metal biodegradation and represents the biodegradation tendency. The corrosion potential of metals can be changed by their internal state such as residual stress, twins and texture [24,25]. A similar corrosion potential in different annealed conditions in this work indicated that they had similar internal state to some extent. Therefore, the non-uniform biodegradation of pure Mg as shown in Figure 7 was attributed to the increase of the grain size, which was consistent with the literature [26] in which the increase of the grain size encouraged higher rates of localized biodegradation and lower rates of uniform biodegradation. For pure Mg in physiological environments, the cathodic reaction is: $2H_{2}O+2e\to H_{2}↑+2{\mathrm{OH}}^{-}$, and the anodic reaction is: $\mathrm{Mg}-2e\to {\mathrm{Mg}}^{2+}$. The obtained biodegradation production, Mg(OH)_2_, had a certain protection for decreasing further biodegradation. In a passive system, grain refinement was documented to decelerate the biodegradation reaction that improved the biodegradation resistance when the biodegradation products on the surface were insoluble or low solubility [27]. In addition, the geometrical mismatch between the oxide layer and the Mg substrate was high because the Piling–Bedworth ratio was only 0.8 [28]. The volume was approximately doubled when the cubic MgO transformed into hexagonal Mg(OH)_2_ in an aqueous solution [29]. The compressive rupture caused by this process led to continual corrosion. The grain refinement can reduce the mismatch stress between the surface layer and magnesium substrate because the fraction of grain boundaries was higher, and accordingly released this stress and decreased the risk of oxide cracking. As a consequence, there was a resulting lower biodegradation rate with a decreasing of the grain size in this work, as mentioned above.

**Figure 8:**
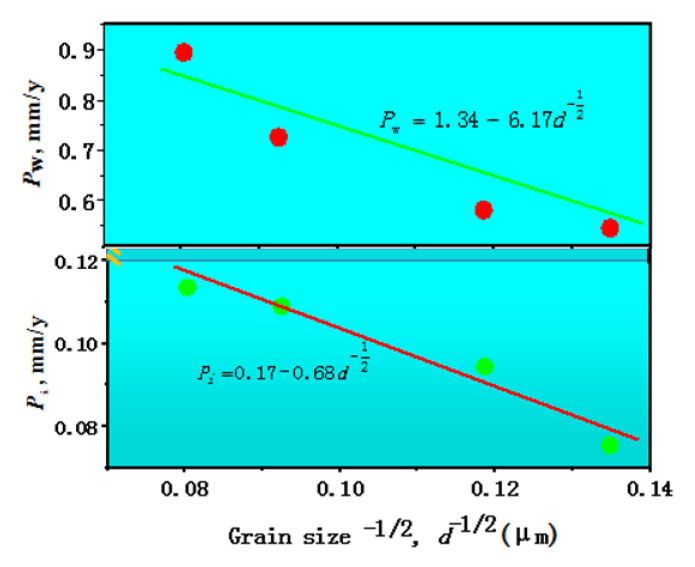
The relation between the biodegradation rate (*P*_i_ or *P*_w_) and the average ferrite size (*d*).

Furthermore, a plot between the biodegradation rate and the minus square root of the grain size, i.e., *P*_i_ versus *d*^−1/2^, or *P*_w_ versus *d*^−1/2^, is depicted in Figure 8, which has a monotonic character. The grain size increased with the increase of the annealing temperature, and the biodegradation rate increased with the increase of the annealing temperature; in other words, with the increase of the grain size, as shown in Figure 8. The *P*_i_ versus d^−1/2^ curve or *P*_w_ versus *d*^−1/2^ synchronized well. This indicated that the biodegradation rate of the pure Mg was grain size-dependent, expressed by a single line that clearly followed a similar Hall–Petch relation. A regression equation to describe the relationship between the biodegradation rate and the grain size was developed as Equation (5) with the correlation coefficient of 0.98 or Equation (6) with the correlation coefficient of 0.96, using all the experimental data.

(5) $$P_{i}=0.17-0.68d^{-}{}^{\frac{1}{2}}$$

(6) $$P_{w}=1.34-6.17d^{-}{}^{\frac{1}{2}}$$

## 5. Conclusions

This work studied the relationship between biodegradation rate and grain size itself, excluding other structural factors such as segregations, impure inclusions, second phase particles, sub-structures, internal stresses and textures caused by alloying additions and deformation processing for pure Mg. The following conclusions can therefore be obtained:

1. Homogeneous microstructures with a spectrum of grain size for pure Mg were obtained by annealing through changing the annealing temperature.
2. The hardness of the pure Mg was grain size-dependent, following a typical Hall–Petch relation: $\mathrm{HV}=18.45+92.31d^{-}{}^{\frac{1}{2}}$.
3. Grain boundary influenced the biodegradation behavior of pure Mg. The biodegradation rate decreased with decreasing grain size, following a similar Hall–Petch relation: $P_{i}=0.17-0.68d^{-}{}^{\frac{1}{2}}$ or $P_{w}=1.34-6.17d^{-}{}^{\frac{1}{2}}$.

## Figures and Tables

**Table 1:** The polarisation data of of the different annealed conditions in Hank’s solution.

Specimens No.	*E*_corr_ (V_SCE_)	*i*_corr_ (μA/cm^2^)	*P*_i_ (mm y^−1^)	*P*_w_ (mm y^−1^)
Mg350	−1.608	3.304	0.0755	0.545
Mg400	−1.596	4.137	0.0945	0.581
Mg450	−1.597	4.414	0.109	0.726
Mg500	−1.598	4.972	0.1136	0.895

**Table 2:** The parameters obtained from the simulation circuit in Hank’s solution.

Specimens No.	R_S_ (Ωcm^2^)	CPEfilm-T (Ω^−1^s^−n^/cm^2^)	*n*	R_f_ (Ωcm^2^)	CPEct-T (Ω^−1^s^−n^/cm^2^)	*n*	R_t_ (Ωcm^2^)
Mg350	19.94	6.963 × 10^−6^	0.706	168.5	20.89 × 10^−6^	0.740	30.42 × 10^3^
Mg400	17.25	11.59 × 10^−6^	0.678	175.0	20.09 × 10^−6^	0.786	16.48 × 10^3^
Mg450	20.50	19.77 × 10^−6^	0.644	178.2	15.97 × 10^−6^	0.807	15.09 × 10^3^
Mg500	19.88	15.04 × 10^−6^	0.665	142.5	15.72 × 10^−6^	0.808	7.257 × 10^3^
